# Understanding abortion-related stigma and incidence of unsafe abortion: experiences from community members in Machakos and Trans Nzoia counties Kenya

**DOI:** 10.11604/pamj.2016.24.258.7567

**Published:** 2016-07-20

**Authors:** Erick Kiprotich Yegon, Peter Mwaniki Kabanya, Elizabeth Echoka, Joachim Osur

**Affiliations:** 1 Ipas Africa Alliance Nairobi, Kenya; 2 Institute of Tropical Medicine, College of Health Sciences, Jomo Kenyatta University of Agriculture and Technology, Nairobi; 3 Kenya Medical Research Institute, Nairobi, Kenya; 4 Amref Health Africa Headquarters, Nairobi, Kenya

**Keywords:** Abortion stigma, Kenya, community, qualitative methods

## Abstract

**Introduction:**

The rate of unsafe abortions in Kenya has increased from 32 per 1000 women of reproductive age in 2002 to 48 per 1000 women in 2012. This is one of the highest in Sub-Saharan Africa. In 2010, Kenya changed its Constitution to include a more enabling provision regarding the provision of abortion services. Abortion-related stigma has been identified as a key driver in silencing women's ability to reproductive choice leading to seeking to unsafe abortion. We sought to explore abortion-related stigma at the community level as a barrier to women realizing their rights to a safe, legal abortion and compare manifestations of abortion stigma at two communities from regions with high and low incidence of unsafe abortion.

**Methods:**

A qualitative study using 26 focus group discussions with general community members in Machakos and Trans Nzoia Counties. We used thematic and content analysis to analyze and compare community member's responses regarding abortion-related stigma.

**Results:**

Although abortion is recognized as being very common within communities, community members expressed various ways that stigmatize women seeking an abortion. This included being labeled as killers and are perceived to be a bad influence for women especially young women. Women reported that they were poorly treated by health providers in health facilities for seeking abortion especially young unmarried women. Institutionalization of stigma especially when Ministry of Health withdrew of standards and guidelines only heightened how stigma presents at the facilities and drives women seeking an abortion to traditional birth attendants who offer unsafe abortions leading to increased morbidity and mortality as a result of abortion-related complications.

**Conclusion:**

Community members located in counties in regions with high incidence of unsafe abortion also reported higher levels of how they would stigmatize a woman seeking an abortion compared to community members from counties in low incidence region. Young unmarried women bore the brunt of being stigmatized. They reported a lack of a supportive environment that provides guidance on correct information on how to prevent unwanted pregnancy and where to get help. Abortion-related stigma plays a major role in women's decision on whether to have a safe or unsafe abortion.

## Introduction

The human toll of unsafe abortion in Africa is the highest in the world, where 56% of the world's unsafe abortions occur, and women face the highest risk of mortality after an unsafe abortion [1]. The World Health Organization estimates that 97% of abortion procedures in Africa are performed unsafely [2], either by an unskilled provider, with inadequate methods or in an unhygienic environment. The poor practice of a safe and common medical procedure results in the death of more than 26,000 African women each year [2, 3]. Most deaths occur where abortion laws are highly restrictive, but they also occur in less restrictive environments. Every year at least 2,600 women die from unsafe abortion in Kenya [4]. The rate of unsafe abortions in Kenya is one of the highest in sub-Saharan Africa region [5] and has increased from 32 per 1000 women of reproductive age in 2002 to 48 per 1000 women in 2012 [6, 7]. In 2012 there was a total of 464,690 induced abortions occurred in both private and Public health facilities [7]. Article 43 of the constitution provides that every person has the right to the highest attainable standard of health, which includes the right to healthcare services, including reproductive health care, Article 26(4) of Kenya's new Constitution allows abortions when pregnancy is a threat to a woman's life or health [8]. Regardless of the legal context, silence and stigma surrounding abortion create an environment in which women make choices that could cost them their lives. Due to lack of understanding of legality and availability of safe abortion services and the stigma associated with abortion, most women resort to unsafe abortion [9]. Kumar et al. argue that a woman who terminates a pregnancy is perceived as having transgressed three conventional ideals of womanhood: female sexuality solely for the means of procreation, the inevitability of motherhood, and instinctual nurturance of the vulnerable and is therefore stigmatized [10]. A lack of knowledge of what is available and what women can legally request, coupled with limited trust in the health system and concerns about cost, cause women to seek unsafe abortions even when safe abortion is legally available [11]. Regional statistics from the APHRC study indicate that different regions in Kenya have different incidences of unsafe abortion, with Rift Valley Region, where Trans Nzoia County is located, reporting the highest incidence of 31 per 1000 women of reproductive age whereas Eastern Region, where Machakos County is located, reporting the lowest incidence of 6 per 1000 women of reproductive age [7]. The goal of this research was to explore abortion-related stigma at thecommunity level as a barrier to women realizing their rights to a safe, legal abortion and compare manifestations of abortion stigma at two communities from regions with thehigh and low incidence of unsafe abortion.

## Methods

**Figure 1:**
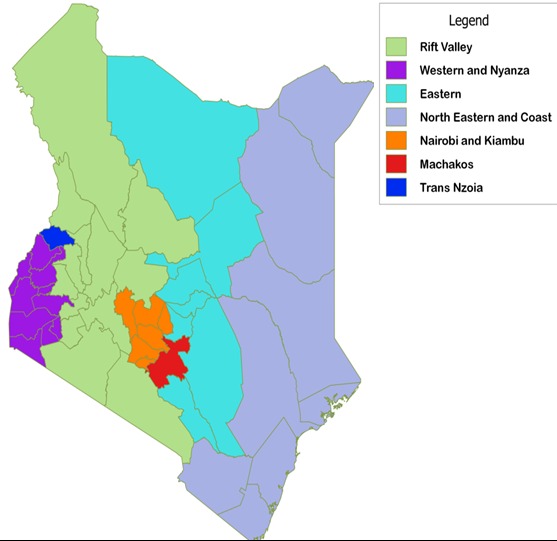
Kenya incidence of unsafe abortion map

We interviewed community members between September and October 2014. We utilized data collected from APHRC study to randomly selected two counties, each from a region with the high and low incidence of unsafe abortion (Figure 1). We then disaggregated each county into three main regions, urban, semi-urban and conducted one FGD targeting, unmarried men, and women, married men and women each conducted separately. This strategy yielded a total of 12 FGDs in Machakos and 14 in Trans Nzoia counties. Table 1 provides background information on the focus group discussions. Using four Community Health Volunteers (CHVs) as per MOH Community Health Strategy [12] in each county we recruited community members from locations where they usually meet for social functions. Such places included churches and water collection points, farms where women were cultivating and saloons separately for unmarried and married men. Unmarried men were recruited from those attending youth activities including video joints, sports events with married men at favorite community clubs, in farms and in men's meetings within the community. The FGD guides covered the following topics: unwanted pregnancy; abortion; community attitudes toward women who have abortions; manifestation of stigma; community views on abortion stigma including women who have undergone an abortion; community views on providers of abortion care services; Community perceptions on women's situation regarding abortion, Sources of information regarding abortion. Data collection team comprising the CHVs underwent two-day training on how to obtain informed consent and in administering the interview guide including using recorders. The FGDs lasted on average two and half hours. All FGDs were recorded using a digital recorder and then transcribed; we then translated all transcriptions into English. The first, second and third authors, read all transcripts in English to identify an initial set of codes. After reading the transcripts, these three members of the team created a codebook of codes and their definitions; it included deductive codes from the guides as well as inductive codes emerging from the data [13]. We uploaded transcripts from all of the FGDs and IDIs into Atlas Ti 7.0 [14]. Each county's set of data was coded by two of the authors. Once all transcripts were coded, we analyzed the codes, grouping them into themes. The unit of analysis was the individual focus group participant. Approval to conduct this study was granted by the Ethical Review Committee of Kenya Medical Research Institute (Scientific Steering Committee No. 2768). Permission to conduct the study at the communities was granted by the County Director of Health in both regions written consent was given by the informants both at the community level. Each respondent was required to sign an informed consent form to participate in the study. Informed consent was obtained by the trained research assistant at a location within the community where the respondent felt it was safe to respond to the questions. No identifier marks or personal information was used in the analysis and subsequent reporting of the study results.

**Table 1:** Number and characteristics of focus group discussions a conducted in each region Kenya

	Machakos	Trans Nzoia
**Timing**	September 2014	October 2014
**Regions**	Eastern region	Rift Valley region
**Sub-regions**	Masinga Sub County Yatta Sub County Kangundo Sub County Matungulu Sub County Kathiani Sub County Machakos Town Sub County Mwala Sub County	Cherangani Sub County Kwanza Sub County Saboti Sub County Endebess Sub County Kiminini Sub County
**Number of groups**	12	14
**Characteristics of Participants-Focus group discussions**	2urban &2 rural 4 20 years or younger 4 24-29 years old	4 urban & 4 rural 3 20 years or younger 3 24-29 years old
**Language of focus group discussion**	Swahili and Kamba	Kalenjin, Luhya and Swahili

## Results

### Community perceptions of women & abortions

When asked how a woman known to have had an abortion or perceived to have had an abortion would be treated by her community, men and women in all the focus groups in both counties discussed the countless ways women are isolated, labeled, and stigmatized. Although abortion is recognized as being very common in their communities, women who have had, abortions are labeled as killers and are perceived to be a bad influence for women, especially young women. Additionally, women are isolated by their peers and considered ‘poor candidates’ for marriage. A young woman in Machakos County illustrates this point and describes how women who have had abortions are perceived by others in the community said: *“They see them as girls with bad habits; they neglect them. Some even say that if you associate with she will teach you bad habits. But the one of being neglected make them feel then even the community does not want them because they never liked what she did.”(Young woman, Trans Nzoia County)*

Unfortunately, due to the fear of being stigmatized, women become fearful of going to the hospital or clinic because of confidentiality issues. As a result, women seek traditional methods of abortion from less skilled and less expensive providers. A young woman describes why women opt for traditional methods: *“Somebody thinks that if I go to the hospital he or she will ask me for so much money, and he or she will also monitor me that now what has this person came to do here, so many people go for the traditional way so that no one knows. It becomes a secret such that you can't tell what is happen to the person (married woman, Machakos County)*

One man from Trans Nzoia County pointed out how the public stigmatization of women who have abortions drives them to seek clandestine procedures: *“We still go after these girls calling them names like, she is useless she is terrible, and this makes the girl hate herself, that's why they hide and even die while aborting.”*

The health risks associated with complications due to unsafe induced abortions was also reiterated in many discussions and served to stigmatize the practice further. One woman in Trans Nzoia County said: *“If one is pregnant, she should keep the baby. Abortion is associated with many problems, one can get an infection, and the uterus gets damaged, this means you cannot have any other child.”*

### Perceptions on abortion & knowledge of the law

When community members were asked how a woman believed to have had an abortion would be treated in their communities, they described various ways that women are ostracized, labeled and stigmatized. Women known to have had an abortion are socially isolated, labeled as killers or murderers, are perceived to be a bad influence, are called prostitutes; women are accused of being unfaithful and younger women are perceived to be poor candidates for marriage. A married woman from Machakos County explains: *“[The woman who aborted] is deemed not to have morals. She is bad company and [the community] will advise [others] not to interact with [her].”* (Married woman, Machakos)

Because of the fear of social isolation, women try to keep the abortion secret and limit the number of people they tell. The fear of social isolation was strong enough to prevent women from seeking safe abortion services, even when services were available in the nearby facilities and had travel to facilities located several Kilometers away from their homes. One old woman reported: *My son is a soldier in Somalia. Given that he was away for a year, my daughter in law became pregnant of another man. She had tried to get rid of the pregnancy quietly so that no one knows. She went to health center [A] to terminate the pregnancy, but no one knew. If we had known, we would have taken her to health facility [B] which is just behind here. She died on her way to health center [A], and she did not have a fare. It was a shame on all of us that we had not assisted her (married woman Trans Nzoia)*

Most community members believed that abortion was illegal or wasn't sure whether it was legal or not. For this reason, women described heightened secrecy around seeking an abortion, both on the part of the woman seeking an abortion and on the part of the provider. In Machakos, County women thought that abortion was not available in the public hospital, but that doctors from public hospitals could provide abortions in private settings if women agreed to keep it secret. One woman explains: *“[Abortion] services are not legal. If you go to a health center for abortion, they will tell you ‘we don't do that.‘ And if they have to do it for you, it's just back door. And the blame is on you.”* (Young woman, Machakos)

The secrecy and known illegality of abortion perpetuate the idea that abortion is an illegal and illicit “back door” activity, which often leads to unsafe abortions.

### Quality of care at public health facilities

In several of the focus group discussions, poor treatment at health facilities as well as a lack of youth friendly services, particularly for unmarried women, became an emerging theme. In Trans Nzoia County, eight focus group discussions with women only compared to three out of twelve focus group discussions in Machakos County reported poor treatment of women seeking abortion by providers in government health facilities. Poor treatment included provider hostility and lack of privacy and confidentiality. Young women reported feeling discouraged and reluctant to go to health facilities because of fear of poor treatment due to their age. One young woman describes this: *Interviewer: “Suppose that you are pregnant and go to [the local] hospital, how will the doctors there receive you?” Participant 1: “They do insult patients. You can go to the hospital and then the doctors’ start talking ill about you, so this discourages you so much, and you decide to leave.” (Young woman, Machakos)*

Healthcare worker attitudes were mentioned as being especially prohibitive to seeking induced abortions at facilities. Both male and female focus group discussion participants shared stories about young women being insulted and chastised for being pregnant. Providers were known for routinely deny young women induced abortion procedures altogether. One woman in Trans Nzoia County said: *“If you are a teenager less than 15 years [old] who is pregnant and have decided to carry out abortion, the midwife will not accept an abortion procedure for you.” (Young woman, Trans Nzoia)*

### Perceptions that no abortion is safe

In both counties, both male and female focus group discussions reported that they did not believe there were any safe abortion methods, and all methods were risky and could lead to death. Six out of twelve focus group discussions in Trans Nzoia County compared with two out of twelve in Machakos County reported that they believed abortion was safer at higher gestational ages. A young woman explained: *“When you look at all the local means used to abort at home, you may end up dying. [It's] the same thing when you go to the hospital, those processes may bring you complications. There are no safe means of abortion.”* (Young woman, Trans Nzoia County)

In Trans Nzoia County, women reported that they would be turned back by Traditional birth attendants to come back later when they thought the “medicine” would work better. If taken earlier in the pregnancy it would lead to death. *My friend who had taken her daughter was advised by the Mkunga to come back later since it is too early for the medicine to work. It is better when the “thing” has formed. It is dangerous when it is too early and also too late. You need the right time*. (Married woman, Machakos County)

### Effects of withdrawal of standard and guidelines by Ministry of Health

Women in Trans Nzoia County noted that there was program addressing sexual and reproductive rights in the community. This program enabled community members to seek SRH services including abortion care services. However, there seems to be a change of heart in the roll out of these services like health facilities that used to provide services ceased offering these services. *Over the last one few months, Community health workers used to refer women for services in the facilities. However this later changed and it is now very hard to obtain these services. I wonder why the change of heart. They used to be very friendly not any more. We are forced to go back to the Wakunga [Traditional Birth Attendants] for help. They are not as good, but they help anyway. (Young unmarried woman, Trans Nzoia)*

Women in Machakos County were not aware of standards and guidelines on managing abortion-related complications from the Ministry of Health (MOH), and none of the groups reported any knowledge of the standards and guidelines. With the passage of the new constitution, the Ministry of health developed standards and guidelines guiding provision of abortion services. However, these standards and guidelines were withdrawn, and therefore health facilities that relied on this guideline stopped offering these services as a result of a change of policy guidelines from the Ministry of Health.

### Cost of abortion care services

The cost of the abortion from any provider was ambiguous and in many instances negotiable and depended on the gestational age of the pregnancy. Secrecy, perceived illegality, higher costs of safer methods and general lack of knowledge about safe abortion methods drive women to untrained providers with lower costs. In both counties it was widely reported that young and poor women had the greatest difficulty accessing the resources necessary to pay for a procedure and had to rely on boyfriends, other friends and mothers, while those with social support from parents, or husbands had greater ability to access or mobilize resources. One woman from Machakos reported: *“For someone to visit the hospital, they get intimidated and can be charged about Ksh.5, 000 [100USD] and beyond. A young teenage school girl may choose to use herbal medicine”*. (Married woman, Machakos)

The perceived high cost of health services was also seen as a barrier to safe abortion. Women also reported not being able to afford the cost accessing drugs for abortion and post-abortion care. Husbands who are often in control of household resources were perceived to be generally unsupportive of abortions as well. One woman who was out of school explained: *“If you don't have 3,000shs to start the service, then you are ignored. If the pregnancy is still young, the cost is between 5,000shs and 8,000shs, but to [get service you need to know [the provider's] name.”* ( married woman, Trans Nzoia)

## Discussion

One of the questions we were most interested in answering in our study was if the manifestation of abortion-related stigma was different in regions with high and low incidence of the unsafe region in Kenya. In both counties the same barriers to safe abortion were shared again and again - fear of being arrested or ostracized for seeking abortion-related information, fear of maltreatment at medical facilities and a lack of knowledge that allows for informed decision-making. While there is a host of other issues potentially at play, the community members who participated in the study suggested abortion stigma and its effects on the dissemination and seeking of abortion-related information is one of the main culprits. A similar story arose in both counties on how someone with an unplanned pregnancy ends up having an unsafe abortion. A clear start to addressing these barriers is the provision of accurate medical and legal information on safe abortion directly to women, especially as part of reproductive health campaigns [15]. Additionally, information needs to be presented and packaged in a way that is simple and clear to a variety of women at different levels of literacy. Withdrawing guidelines for health providers to offer high quality of care on abortion further stigmatizes abortion, increases the likelihood that women will make an uninformed and unsafe choice, and makes existing efforts to provide women with reproductive health knowledge a missed opportunity for saving the lives of the 47,000 women who die of unsafe abortion each year [1]. In each county, women said they want to receive information about abortion from the same people who explain their family planning options-for most of these women the person delivering this information was a Community Health Volunteer [16]. At a minimum, they need to know that safe abortion and unsafe abortion are very different things and under what circumstances abortion is legal and available in their country. Equipped with this knowledge, they have a reason to seek out one kind of abortion over another and the knowledge that while asking about abortion may cost them status in their community it will not mean that they go to jail. If CHVs and providers in facilities simply knew the information and shared accurate information on abortion without shaming those who ask for it, this would be a huge step in the right direction. When MOH issued a circular withdrawing standards and guidelines for the provision of abortion services, this was generally interpreted to mean that abortion services should not be provided, and this further went to increase how abortion is stigmatized. Community members reported that Health providers were afraid of offering them services and therefore referred them to seek services outside health facilities as they did not want to be associated with abortion.

## Conclusion

Abortion is a highly stigmatized subject in communities. However, communities in Trans Nzoia County expressed more stigma compared to women in Machakos County, especially for young unmarried women. With Ministry of Health withdrawing standards and guidelines that offered opportunities for women to seek safe abortion services, women were left to seek abortion services in unsafe places. Stigma is an important contribution on whether or where a woman will seek a safe or unsafe abortion, eventually increasing incidences of unsafe abortion, if options available are stigmatizing to the woman. In order to address the challenge of unsafe abortions, initiatives targeting stigma reduction interventions both at the health facility and community level must be put in place.

### What is known about this topic

- Unsafe abortion is one of the key drivers to maternal mortality in Kenya contributing to a third of all maternal deaths and disabilities;
- The incidence of unsafe abortion in Kenya increased from 32 to 48 per 1000 women of reproductive age in 2002 and 2012 respectively;
- Women seeking abortion or anyone associated with the provision of unsafe abortion is usually stigmatized.

### What this study adds

- Despite passing a new constitution in 2010 that enhances women's reproductive rights, most community members do not know these rights including how to exercise these rights;
- When MOH withdrew standards and guidelines on provision of unsafe abortion at health facilities in 2013, women seeking abortion services were driven to seek unsafe abortion services for fear of being stigmatized;
- Community members from regions with high incidence of unsafe abortion narrated instances where a woman seeking unsafe abortion would be more stigmatized compared to women from regions with low incidence of unsafe abortion.
